# Counselling on Conceiving: Attitudes and Factors Influencing Advice of Professionals in Transplantation

**DOI:** 10.3389/ti.2023.11052

**Published:** 2023-05-10

**Authors:** Marleen C. van Buren, Margriet Gosselink, Emma K. Massey, Jacqueline van de Wetering, A. Titia Lely

**Affiliations:** ^1^ Department of Internal Medicine, Erasmus MC Transplant Institute, University Medical Center Rotterdam, Rotterdam, Netherlands; ^2^ Department of Obstetrics and Gynaecology, Wilhelmina Children’s Hospital Birth Centre, University Medical Center Utrecht, Utrecht, Netherlands

**Keywords:** kidney transplantation, nephrological care, pregnancy, counselling, gynaecologist

## Abstract

Pregnancy after kidney transplantation (KT) conveys risks of adverse pregnancy outcomes (APO). Little is known about performance of pre-pregnancy counselling after KT. This study investigated perceptions of risk, attitudes towards pregnancy and factors influencing advice given at pre-pregnancy counselling after KT. A web-based vignette survey was conducted among nephrologists and gynaecologists between March 2020 and March 2021, consisting of five vignettes containing known risk factors for APO and general questions on pre-pregnancy counselling after KT. Per vignette, attitudes towards pregnancy and estimation of outcomes were examined. In total 52 nephrologists and 25 gynaecologists participated, 56% from university hospitals. One third had no experience with pregnancy after KT. All gave positive pregnancy advice in the vignette with ideal circumstances (V1), versus 83% in V2 (proteinuria), 81% in V3 (hypertension), 71% in V4 (eGFR 40 ml/min/1.73 m^2^). Only 2% was positive in V5 (worst-case scenario). Chance of preeclampsia was underestimated by 89% in V1. 63% and 98% overestimated risk for graft loss in V4 and V5. Professionals often incorrectly estimated risk of APO after KT. As experience with pregnancy after KT was limited among professionals, patients should be referred to specialised centres for multidisciplinary pre-pregnancy counselling to build experience and increase consistency in given advice.

## Graphical Abstract

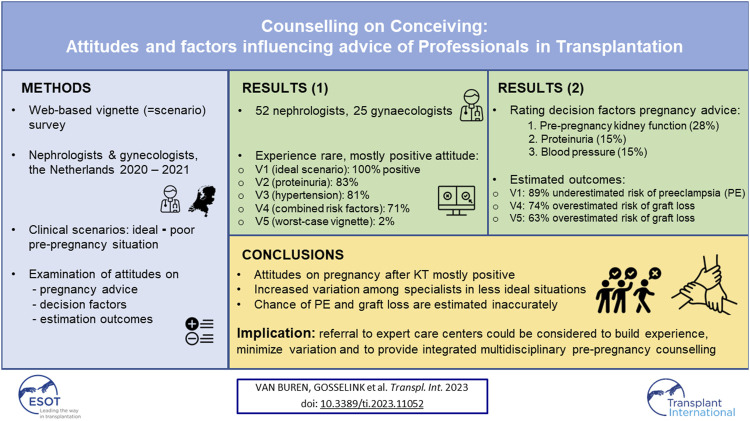

## Introduction

Pregnancy after kidney transplantation (KT) is challenging from both an obstetric and renal point of view. Higher incidences of adverse pregnancy outcomes (APO) have been described, such as preeclampsia, foetal growth restriction and preterm birth (1–3). Pregnancy does not seem to negatively affect graft function or graft loss when pre-pregnancy kidney functioning is good (4).

Since the first successful pregnancy after KT in 1958 (5), annual numbers of pregnancy after KT have been rising. In the US, on average 220 women conceive and give birth after KT per year, in the Netherlands on average 12 women per year (6).

Although challenging, women after KT have the same desire for children and often have considered their plans for pregnancy to a greater extent than women in the healthy population (7, 8). Therefore, pre-pregnancy counselling is an important aspect of clinical care for kidney transplant recipients. Our previous study showed that kidney transplant recipients rely on their nephrologists’ pregnancy advice and that attitudes towards pregnancy vary between nephrologists (9).

According to the best practice guidelines from 2002 (European) and 2005 (United States) (10, 11) optimal timing of pregnancy after KT is at least 1–2 years after transplantation, in women with good kidney function, little/no proteinuria, normal blood pressure, no recent acute rejection, good compliance to medication and no use of teratogenic drugs. When the situation does not meet these criteria, practice guidelines advise evaluation on case-by-case basis.

While guidelines describe the ideal candidate for pregnancy after KT, little is known about pregnancy in less ideal situations. Furthermore, physicians do not always follow clinical practice guidelines (12, 13). This cross-sectional survey vignette study was designed to examine the variation in attitude of medical specialists regarding pregnancy after KT in varying situations. Also, factors influencing their attitude and pregnancy advice were examined.

## Materials and Methods

### Study Design

A cross-sectional survey vignette study (14–17) was conducted between March 2020 and March 2021. To determine variation in pre-pregnancy counselling between medical specialists in the Netherlands, five clinical vignettes were constructed. Participants were invited by e-mail to complete a web-based questionnaire concerning these vignettes. LimeSurvey software was used to create the survey and collect data (18). The Checklist for Reporting Results of Internet E-Surveys (CHERRIES) was used for reporting the results of our study (Supplementary File S3) (19).

### Participants

Nephrologists and gynaecologists practicing in public hospitals were invited to participate. Of note, post-KT care in the Netherlands is mainly carried out by university medical centres during the first year after KT. After 1 year, patients are referred to general hospitals for further care. Therefore, patients with a wish to conceive may be undergoing treatment either in university or in general hospitals. To enable inclusion of participants in both settings, the survey was sent to the regional network of the research group. Participants were invited by an initial e-mail to fill in the questionnaire, followed by two reminders. Responses were also included if the questionnaire was not fully completed. The survey was only accessible for the invited participants and was protected by a password.

### Vignettes

Vignette studies use short scenarios (vignettes) for respondents in surveys to express their views and attitudes on these scenarios. By systematically varying the levels of theoretically important vignette characteristics, a sample of different vignettes is available for respondents to judge (17).

For our study, vignettes were carefully constructed according to several steps. First, vignettes were designed based on previous literature and clinical expertise (4). (1, 20, 21) Then, vignettes were evaluated by two experienced specialists in counselling for pregnancy after KT: one obstetrician and one transplant nephrologist. The vignettes were then reviewed by a health psychologist involved in survey research, to check for clear wording and corresponding questions and answer categories. Finally, a study pilot was conducted by sending the survey to three transplant professionals to test understanding and acceptability. According to these responses, the vignettes and questions were revised.

The vignettes described the same case of a woman of reproductive age after KT, coming to the outpatient clinic with a wish to conceive. In each vignette, one factor was adjusted to assess factors influencing attitudes towards pregnancy and advice. Although the decision making process is complex and multifactorial, only the most important risk factors for adverse pregnancy outcome (1, 2, 4, 22) could be included in this study because of the expected number of respondents. Vignettes varied on the following characteristics: presence of hypertension (blood pressure >140/>90 mmHg), proteinuria (>500 mg/L), poor kidney functioning (eGFR <60 mL/min/1.73 m^2^) and rejection in the past year (21). The first vignette described the ideal situation for pregnancy after KT, with no risk factors for poor outcomes. The second to fifth vignette introduced, respectively proteinuria, hypertension, poor kidney function (eGFR 40 mL/min/1.73 m^2^) and a combination of risk factors (hypertension, eGFR 25 mL/min/1.73 m^2^, proteinuria, rejection in the past year). In the supplementary data file, the vignettes and questionnaire are shown (Supplementary Files S1, S2).

### Survey

The survey consisted of three parts: first, questions regarding participants’ characteristics and their experience with pregnancy after KT. Furthermore questions were asked about counselling style and responsibility. Additionally, participants were asked to rank the factors that influence their advice regarding pregnancy from a scale of 1–5 (Likert scale). These factors were identified from current literature (1, 4, 10, 11) (Supplementary File S1). Second, vignettes were displayed and per vignette, participants were asked whether their attitude towards a pregnancy for this patient would be negative or positive. Also, the weight of decision factors for their attitude was examined (on a scale of 1–5). Furthermore, participants had to predict the pregnancy outcome of the given vignette with respect to gestational age, birth weight, chance of developing preeclampsia and chance of graft loss within 2 years after pregnancy. Lastly, participants were asked to name and rate (on a scale of 1–5) the most important factor influencing pregnancy advice after KT per vignette (Supplementary File S2).

### Ethics

There were no patients involved in this study. Personal information of participants was pseudo-anonymized. Data was collected and stored in a secured database. The study was approved by the Ethical Committee of the Erasmus Medical Centre: MEC-2020-0194.

### Analytical Approach

Continuous values are reported as means (SD) when they were normally distributed. Variables with a non-normal distribution are reported as median with interquartile range (IQR). For each vignette, positive and negative attitudes towards pregnancy were analysed. Per vignette, the study group was divided into a positive attitude group and negative attitude group and groups were cross-tabulated against participants’ demographic characteristics. Also, per vignette, participants’ estimated outcomes were compared with observed pregnancy outcomes after KT in the PARTOUT-dataset, in which all pregnancies after KT and their outcomes of the past 40 years in the Netherlands are included (22). Unfortunately, only for vignette 1, 4 and 5 a comparison with current literature could be made since for vignette 2 (proteinuria) and vignette 3 (hypertension) no comparative data were available. Furthermore, a ranking was made per specialty for factors influencing pregnancy counselling and advice. Significance between groups was determined by a T-test or Chi-square test. Significance was corrected for multiple testing with the Bonferroni correction (23). Analyses were performed using IBM SPSS Statistics, version 25.0.0. Graphs and figures were established with GraphPad Prism, version 8. Free text-responses were categorized.

## Results

### Participant Characteristics

**FIGURE 1:**
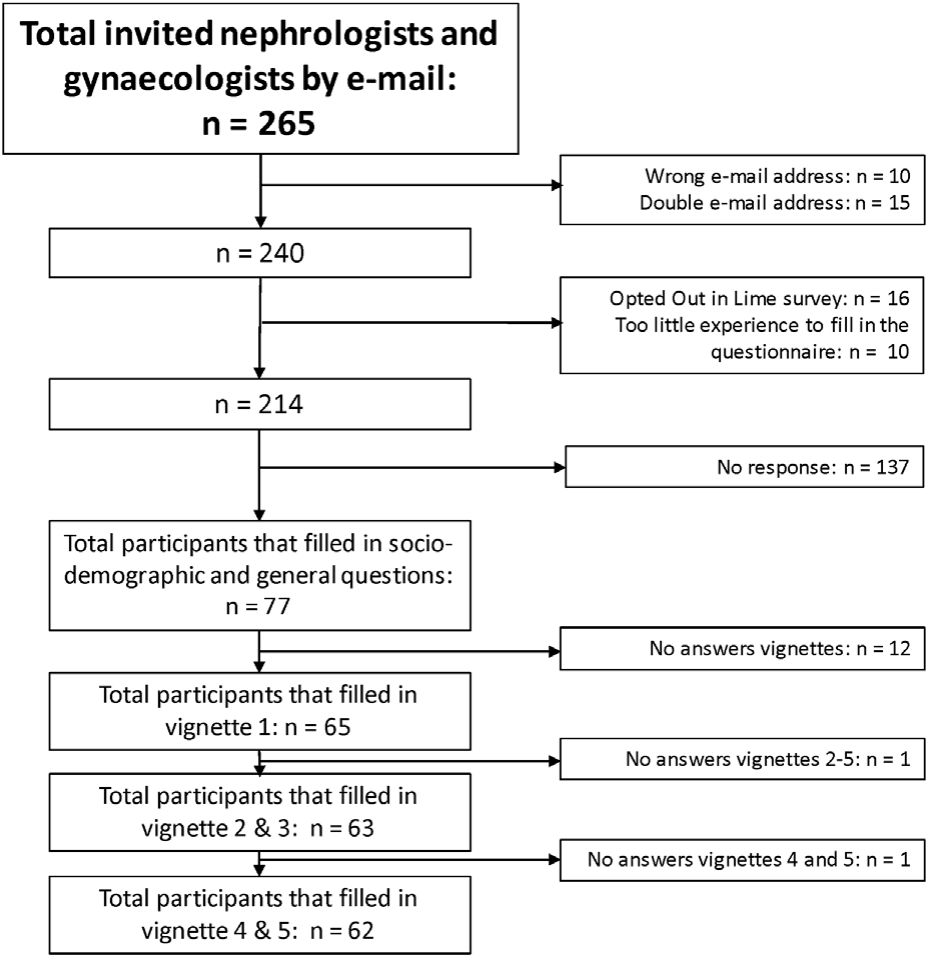
Flowchart of this study. In total, 77/240 (32%) participants replied to the questionnaire.

In total, 265 medical specialists were invited to participate in this questionnaire. After removal of non-existing and duplicate email addresses and participants that opted out, 77/240 participated (32% response rate, Figure 1). Participant characteristics are reported in Table 1. The study group consisted of 52 (68%) nephrologists and 25 (32%) gynaecologists.

**TABLE 1:** Participants’ baseline characteristics.

Demographic variable	N = 77 (n/%)
Medical centre	
- University Hospital	43 (56%)
- General Hospital	34 (44%)
Function	
- Gynaecologist	25 (33%)
- Nephrologist	52 (68%)
Year of graduation medical training (median, IQR)	2006 (10)
Age (IQR) (median, IQR)	47 (13)
Gender	
- Male	39 (51%)
- Female	38 (49%)
Children of their own	71 (92%)
Dutch nationality	76 (99%)
Religion, of which:	17 (22%)
- Christianity	16 (21%)
- Islam	1 (1%)
Working experience in KT, years (median, IQR)	12 (14)
Number of women with pregnancy after KT treated by the participant (median, IQR)	3 (15)

KT, kidney transplantation; IQR, inter quartile index.

### Experience and Opinions on Counselling

#### Experience With Pregnancy Outcomes

Overall, 76/77 of participants answered the question regarding their experience of treating women who became pregnant after KT. The majority (57%) reported good experiences with pregnancy after KT. 35% of respondents indicated having too little experience with pregnancy after KT to answer this question. Furthermore, participants were asked to clarify their definition of good pregnancy outcomes. Regarding the child, answers varied from being “born at term” to “birth after 36 weeks without complications for the child or growth retardation.” Regarding the mother, quotes varied from “birth without complications” to “stable graft function, uncomplicated pregnancy and being able to enjoy the pregnancy and birth.” Regarding the graft, definitions of poor outcome ranged from “decline in eGFR” to “renal replacement therapy.” One nephrologist stated: “transplant survival is not the only important outcome in life.” Another stated: “Pregnancy after transplantation is not a pink cloud, but a medical obstacle course where parents should make a conscious decision. But if you make this choice with the right guidance, the outcome can be successful.”

#### Counselling Style

Participants were questioned about their counselling style. Most participants responded to be more informing and coaching than directive: “Informing, but more guiding when there are great risks.” Also, participants indicated that styles differed per type of patient.

#### Responsibility of Decision Making

The majority of participants see themselves as “responsible” to “very responsible” in the decision-making process when a patient is wishing to conceive after KT (64%). However, 8% felt no responsibility “as long as the patient is not in need of assisted fertility, she alone is responsible” and 29% felt little responsibility “it is the decision of the patient, it is her life.” Regarding responsibility for a pregnancy, most participants indicated that the professional/clinician is responsible for informing the patient about several scenarios of outcomes. The responsibility for the final decision to conceive lays with the patient: “The doctor advises, the patient decides, always.” Also, a difference was made in spontaneous conception versus assisted pregnancies, “if there is enough proof for a negative medical pregnancy advice then you should have the guts to offer no fertility treatments, that is really a responsibility of the doctor.” Only a few participants (two nephrologists) thought that the clinician was responsible for the final decision, because of his/her medical expertise.

#### Factors Influencing Counselling Advice

**FIGURE 2:**
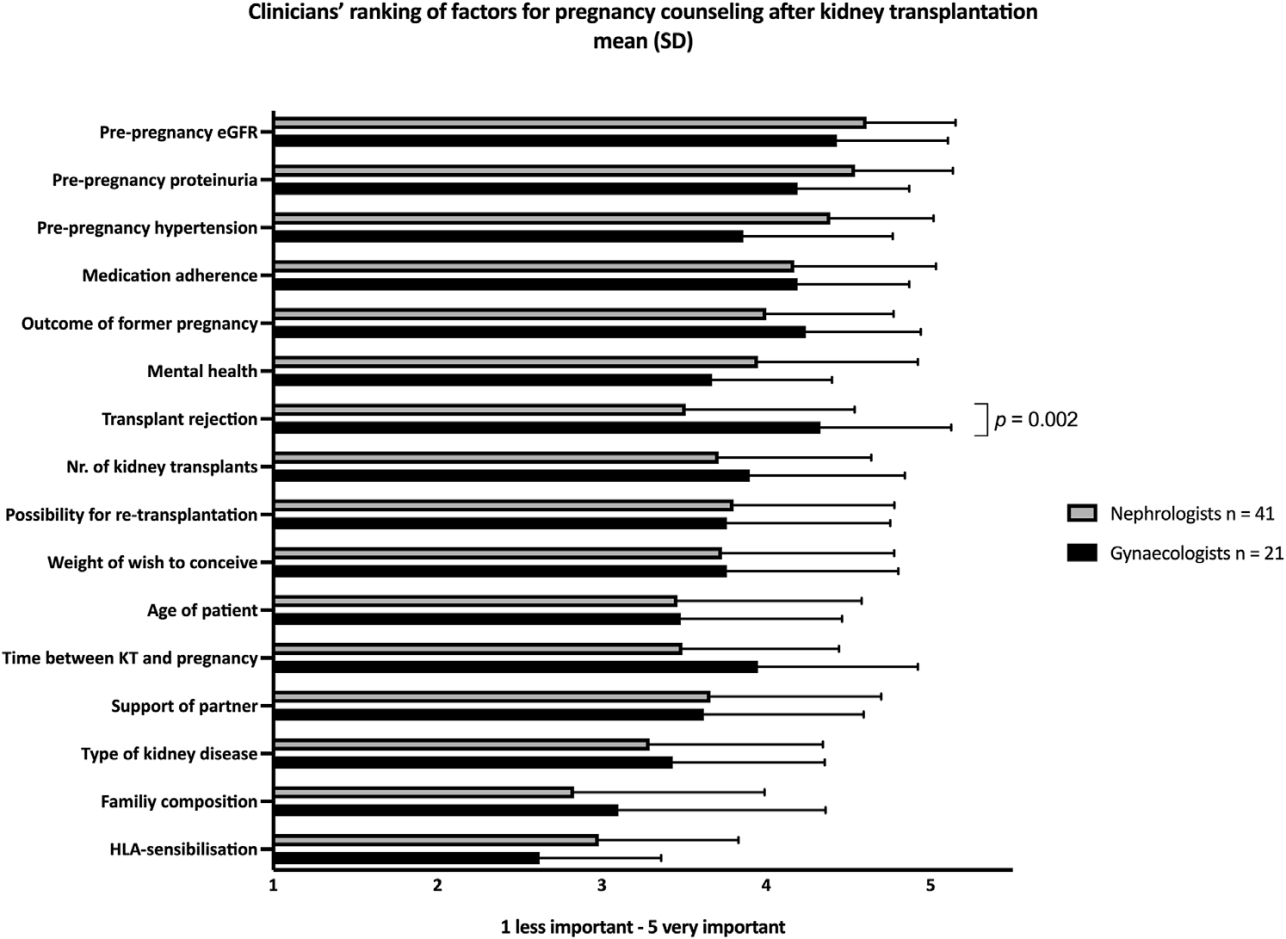
Importance of factors in pre-pregnancy counselling after kidney transplantation in general, according to clinicians. Scale 1 (less important) to 5 (very important). Gynaecologists scored “history of transplant rejection” significantly more important than nephrologists (*p* = 0.002). Significance was determined by T-test. The use of Bonferroni’s post-test correction adjusted significance level to (0.05/16) *p* < 0.0031.

The results are shown in Figure 2. Gynaecologists ranked “graft rejection in the past” significantly more important than nephrologists (*p* = 0.002).

Furthermore, participants were asked to rank their three most important factors for pre-pregnancy counselling andadvice. Of all these factors, pre-pregnancy eGFR was considered most important (28%), followed by pre-pregnancy proteinuria (15%) and pre-pregnancy blood pressure (14.5%). Co-morbidity, obstetric history, mental health, smoking, BMI, attitude towards potential adverse pregnancy outcomes were also factors that were taken into consideration.

### Vignettes

**FIGURE 3:**
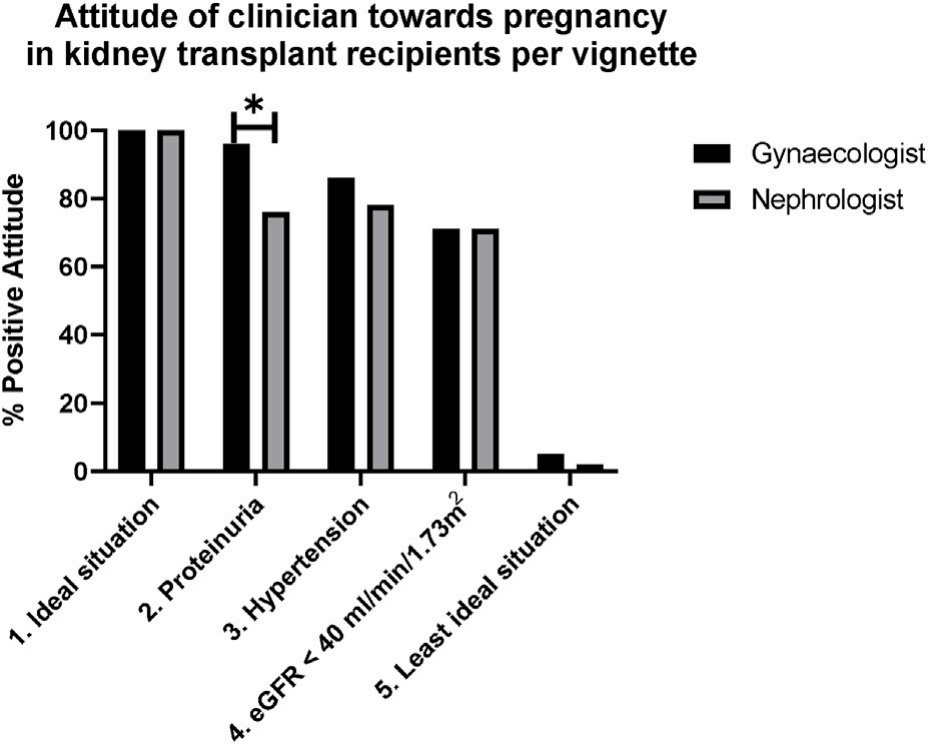
Attitudes of clinicians towards pregnancy in kidney transplant recipients per vignette. *In Vignette 2 (proteinuria), gynaecologists were more positive than nephrologists, *p* = 0.045 considered not significant. Significance was determined by Chi-square. The use of Bonferroni’s post-test correction adjusted significance level to (0.05/5) adjusted the significance level to *p* < 0.01.

For each vignette, the number of positive attitudes towards pregnancy after KT is shown in Figure 3.

In the first vignette (ideal situation), all participants had a positive attitude towards pregnancy after KT. In the second vignette (proteinuria) 83% was positive. As shown in Figure 3, while more nephrologists (10/41, 24%) had a negative attitude than gynaecologists (1/22) (*p* = 0.045), this difference was not significant. Reasons for negative advice included: “risk of graft failure,” “examine reason for proteinuria before getting pregnant” or “inform patient regarding high risk of graft failure and preeclampsia.” In the third and fourth vignette (hypertension and poor kidney function), respectively 81% and 71% were positive. In the last vignette (worst case), 98% of participants had a negative attitude towards pregnancy. A nephrologist stated “do not become pregnant, unless the woman is of higher age and is not able to wait any longer, and only if she knows this could lead to the loss of her kidney graft.” The one gynaecologist who would give positive advice for this vignette explained: “in the end it is a patient’s choice, but counselling should be very attentive with all concerns thoroughly explained: it will be a high-risk pregnancy with high chance of complications.” No significant associations were found between demographic characteristics and attitude towards pregnancy.

#### Estimated Outcomes of the Vignettes

**FIGURE 4:**
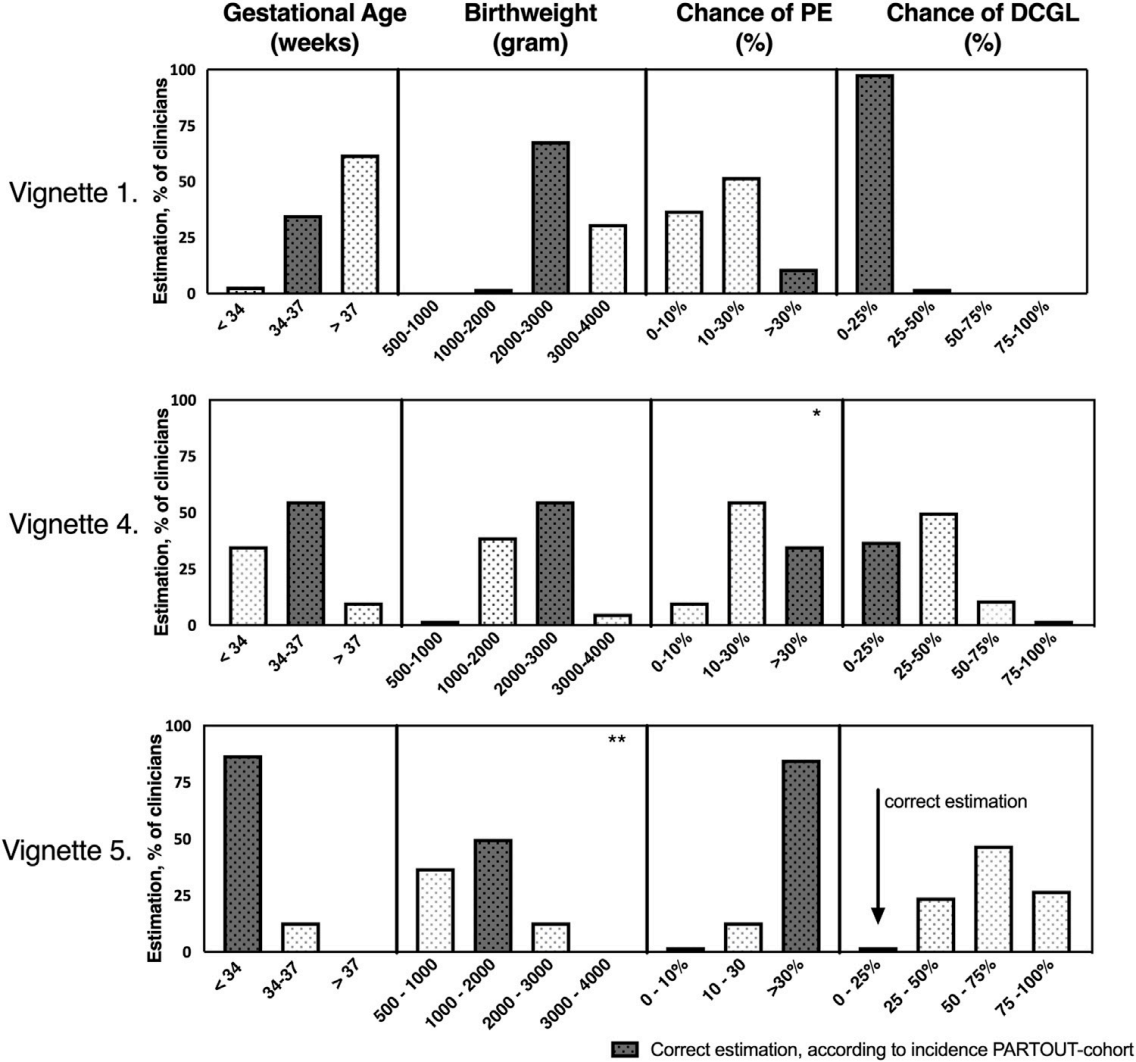
Clinicians’ estimations regarding pregnancy outcomes per vignette compared to the true incidences of pregnancy outcomes in the PARTOUT-cohort. Vignette 1 = ideal situation eGFR 65 ml/min/1.73 m^2^ (*n* = 65). Vignette 4 = eGFR <40 ml/min/1.73 m^2^ (*n* = 62). Vignette 5 = least ideal situation (combination of factors), eGFR 25 ml/min/1.73 m^2^ (*n* = 62) For each vignette, the dark bar represents the correct estimation according to the PARTOUT-dataset. PE: preeclampsia, DCGL: Death Censored Graft Loss within 2 years postpartum. * Chi-square: *p* = 0.004 nephrologists estimated lower chance of developing preeclampsia than gynaecologists. ** Chi-square: *p* = 0.020 nephrologists estimated higher birthweight than gynaecologists. Both considered not significant. The use of Bonferroni’s post-test correction adjusted significance level to (0.05/20) *p* < 0.0025.

In Figure 4, for vignette 1, 4 and 5, participants’ predictions of outcomes are shown, compared to the observed outcomes in the PARTOUT-dataset and current literature. The dark grey bars are the “true” results from the PARTOUT dataset (22).

In vignette 1, the majority (62%) predicted a higher gestational age than observed in the PARTOUT-dataset (estimated >37 weeks versus mean gestational age PARTOUT-data 36 weeks). In vignettes 4 and 5, estimated birthweight corresponded with the PARTOUT-data. The chance of developing pre-eclampsia was underestimated in vignette 1 (ideal situation): 89% of participants estimated the chance of preeclampsia <30% while the PARTOUT-dataset showed an incidence of 39%. Estimated outcomes of vignette 2: proteinuria, and vignette 3: uncontrolled hypertension could not be compared with current literature, since no comparative data on these parameters were available. Although the difference was smaller, in vignette 4 (eGFR 40 mL/min/1.73 m^2^) the incidence of preeclampsia was underestimated as well (estimated 10%–30% versus PARTOUT-data 33%–39%). The chance of graft loss was overestimated in vignette 4 (eGFR 40 mL/min/1.73 m^2^) and 5 (worst case) by respectively 63% and 98% of participants.

Lastly, predicted outcomes were compared between nephrologists and gynaecologists. After adjusting for multiple testing, *p* < 0.0025 was considered significant. In vignette 5 (worst case), nephrologists predicted a higher birth weight than gynaecologists (*p* = 0.046, *p* = 0.020). Furthermore, nephrologists estimated a lower chance of developing preeclampsia than gynaecologists in vignette 4 [eGFR 40 mL/min/1.73 m^2^ (*p* = 0.004)]. These differences were not significant after adjusting for multiple testing. No association was found between years of experience and prediction of outcomes.

## Discussion

### Main Conclusion

This study, focusing on attitudes among professionals towards pregnancy after KT, has four major findings. First, professionals had little experience with pregnancy after KT. Among those with experience, attitudes towards pregnancy after KT were positive. Second, pre-pregnancy kidney function, proteinuria and blood pressure are considered most important factors influencing pregnancy advice after KT. Third, despite participants’ overall positive attitude towards pregnancy after KT, in less ideal situations, there was less agreement on pregnancy advice. Fourth, participants seem to underestimate the chance of developing preeclampsia and overestimate the chance of graft loss within 2 years after pregnancy. As pregnancy after KT is rare, referral to expert care centers could be considered to build experience and to provide combined pre-pregnancy care and counselling by a nephrologist and gynaecologist together.

### Comparison With Current Literature

In the Netherlands, the incidence of pregnancy in women who are transplanted under the age of 45 is approximately 10% (9). Therefore, pregnancy after KT is a rare phenomenon in daily practice, especially for nephrologists and gynaecologists in general hospitals. This was also shown in our study, with 30% of clinicians having no experience with pregnancy after KT. Nevertheless, these clinicians can also be confronted with questions regarding pregnancy from KT recipients in daily practice when patients express their wish to conceive to their treating physician. When experience is lacking, clinicians need to turn to guidelines and consensus statements. Unfortunately, these guidelines describe only ideal situations (10, 11). This makes it difficult to counsel more complex cases such as patients with some proteinuria and/or lower kidney function. Furthermore, a previous study among CKD-patients regarding fertility care showed a relationship between knowledge of clinicians on fertility care and the amount of fertility care that was given (24). From this study, it can be hypothesized that with little experience, a clinician might be less attentive to the subject of pregnancy after KT. This may also help explain why, in our previous study, women after KT reported a lack of initiative among clinicians to broach the subject and experienced a high threshold to discuss their wish to conceive with their nephrologist (9).

The ranking of kidney function, proteinuria and blood pressure as the three main important factors for counselling and for risk identification matches current literature and guidelines (1, 4, 10, 11). Clinicians’ estimations were compared to the Dutch PARTOUT-cohort for two reasons. First, to ensure a representative comparison of estimations and reported outcomes on a national scale. Second, to ensure an optimal comparison given the availability of many vignette-parameters in the PARTOUT-data that were lacking in other published cohorts. In this comparison, the chance of developing pre-eclampsia and preterm birth was underestimated by clinicians (22). However, when comparing clinicians’ estimations regarding preeclampsia to outcomes reported in the study by Stoumpos et al, clinicians’ estimations seem more adequate. Nevertheless, Stoumpos’ incidence of preterm birth (61%) was similar to the PARTOUT-dataset (25). Also, the cohort of KT-pregnancies reported by Piccoli et al could be matched to vignette 1 (ideal situation), showing a higher incidence of preterm birth than the PARTOUT-data (26). Thereby, when comparing clinicians’ estimations regarding preterm birth internationally to different cohorts, their predictions remain an underestimation. Unfortunately, to our knowledge, other estimated parameters such as risk of graft loss within 2 years after delivery were not available in other published cohorts for vignette-comparison.

We reported an overestimation of the risk of graft loss within 2 years after delivery compared to the PARTOUT-cohort. There is a relationship between kidney function and the risk for graft loss (27), but in our recent study there was a small but non-significant difference in eGFR-slope before and after pregnancy (28). Additionally, a recent meta-analysis showed no difference in graft loss between women with and without pregnancy after KT (4, 25). Unfortunately, literature on proteinuria and pregnancy outcomes after KT is lacking.

The majority of clinicians had a positive attitude towards pregnancy after KT. This contrasts earlier studies on pre-pregnancy counselling among KT recipients, where respectively one-third and a quarter of female KT recipients reported to have been counselled against pregnancy (29, 30). While the intentions of these clinicians remain unknown, their opinion counts and negative information can be overwhelming for women. It is important that clinicians are aware of their influence and that they have adequate counselling skills. Even a negative tone might lead to cancelling pregnancy plans. Wiles et al also investigated pre-pregnancy counselling in CKD-patients. They found that the clinicians’ positive or negative attitude towards pregnancy had an influence on the decision to become pregnant (31). Taking this influence into account, it is desirable that clinicians are well informed on most recent findings and have up-to-date knowledge on this subject.

Of note, though this study focuses on counselling KT recipients who want to become pregnant, some KT recipients get pregnant without planning. While in Netherlands termination of pregnancy at an early stage of pregnancy is legal, this is not the case in all countries. A recent editorial on the impact of the reversal of *Roe v. Wade* in the United States, further emphasized the importance of reproductive care and pre-pregnancy counselling for women with CKD in countries or states where abortion is not legal (32).

Based on our findings we recommend that in more complex clinical cases pregnancy counselling and care should be carried out in multidisciplinary teams with an individualised approach for the patient wishing to conceive. This is in line with the previous advice by Cabiddu et al. regarding pregnancy after KT in less ideal situations (33).

### Strengths and Limitations

To date, this is the first study investigating attitudes and factors influencing pre-pregnancy counselling after KT among nephrologists and gynaecologists. Another strength is the elaborate and methodical vignette construction. With expertise from experienced transplant professionals (a nephrologist and a gynaecologist), a health psychologist and a pilot study, vignettes were improved and refined. Therefore, vignettes were constructed that fit the research questions. However, despite these efforts, feasibility of the survey required simplification of scenarios and options for advice. Therefore, the fictitious vignettes did not cover the full range of complex dilemmas, possible factors influencing counselling and advice in daily practice. To address this limitation, factors -based on current literature and expert opinion-were ranked by participants next to the vignettes. Another limitation is the low participation rate (32%). This is in all likelihood because pregnancy after KT is highly specialised care. The questionnaire was sent to all nephrologists and gynaecologists in the regional network of the PARTOUT-network. Part of the invitees might not have felt compelled to participate in this study because they were lacking experience with pregnancy after KT. This could have led to selection bias. Although the relationship between prediction of pregnancy outcomes and clinicians’ experience in the transplant field or with pregnancy after KT seems intuitive, this could not be demonstrated. A possible explanation might be the relatively small sample size causing low statistical power. Despite considerable limitations, this study is unique and can contribute to a broader focus on how pre–pregnancy counselling should be performed.

### Implications and Further Research

In order to promote informed shared-decision making, more information needs to be available for patients and clinicians. With outdated guidelines, providing accurate information to the patient is a challenge. On top of this, although the number of women getting pregnant after KT is rising, yearly numbers are still relatively low. Though the recent publication of the PARTOUT-data will assist in counselling for pregnancy after KT, larger international datasets on pregnancy outcomes are needed. Furthermore, to capture the different attitudes in the dilemmas of daily practice more thoroughly, this study could be expanded internationally, to evaluate additional factors that may influence counselling to the vignettes. Although this study does not directly demonstrate experienced professionals predicting pregnancy outcomes more accurately, we suggest pre-pregnancy counselling to be centralized in specialised centres for multidisciplinary pre-pregnancy counselling. This in order to build experience as pregnancy after KT is scarce and often complicated.

## Data Availability

The raw data supporting the conclusion of this article will be made available by the authors, without undue reservation.

## Abbreviations

APO, adverse pregnancy outcomes; BMI, body mass index; CI, confidence interval; CKD, chronic kidney disease; eGFR, estimated glomerular filtration rate; IQR, interquartile range; KT, kidney transplantation; SCr, serum creatinine; SD, standard deviation.
