# Supplementary material for: Counselling on Conceiving: Attitudes and Factors Influencing Advice of Professionals in Transplantation
Source: Transpl Int. 2023 May 10;36:11052. doi: 10.3389/ti.2023.11052 (PMC10205991; doi:10.3389/ti.2023.11052)
Supplement: Supplementary file 1 [file DataSheet1.docx]

**Supplementary Data**

**S1: General Questionnaire**

*Demographic Questions*

1. In what kind of hospital do you work?

*University hospital / general hospital*

2. What is your medical specialty?

*Gynaecologist/nephrologist/ Other…*

3. When did you finalize your medical education? (Year)

4. What is your age?

5. Are you a man or a woman?

6. Do you have children yourself?

*yes/no*

7. Are you religious?

*Nee/Christian/Muslim/Bhudism/Other*

8. How many years of working experience do you have within the field of transplant?

9. In what number of women with a pregnancy after kidney transplantation were you involved in the treatment?

10. In what number of women with a pregnancy after kidney transplantation did you have a leading role?

11. Is your experience with pregnancy after kidney transplantation in general good or poor regarding maternal and fetal outcomes?

*Predominantly good/Predominantly poor*

12. Can you explicate what you consider to be a good pregnancy outcome regarding:

*a. Child*

*b. Mother*

*c. Transplant*

13. Can you explicate what you consider to be a poor pregnancy outcome regarding:

*a. Child*

*b. Mother*

*c. Transplant*

14. In general, do you have a more informing or steering role in counseling women with a pregnancy wish after kidney transplantation?

15. Who is responsible for the decision to become pregnant after kidney transplantation? Please give a score on a scale of 1 (no responsibility) to 5 (most responsibility).

*a. Physician 1-5*

*b. Patient 1-5*

16. Can you give arguments for your choice above?

*General questions*

1. What is your general opinion on pregnancy after kidney transplantation? Do you have a positive or negative stance? Please give a score on a scale between 1 (negative) to 5 (positive)
2. To what extent do you weigh the following factors in your final advice for women who wish to become pregnant after a kidney transplant? Rate each factor from not important (1) to very important (5).

- *Age of the patient*
- *Type of kidney disease*
- *Outcomes of earlier pregnancies*
- *Rejection in this kidney in the past*
- *Amount of time after kidney transplantation*
- *Bloodpressure*
- *Proteinuria*
- *Preconceptional eGFR*
- *Medication adherence*
- *HLA-sensibilisation, percentage PRA*
- *Possibility for retransplantation*
- *Current family composition*
- *Weight of wish to conceive*
- *Support of partner*
- *Nr. of kidneytransplants*
- *Mental health*

1. Which of the three factors above do you consider most important?
2. Why these three factors?
3. Are there any other factors you discuss in your counseling by a patient with a pregnancy wish? (that are not mentioned earlier?)
4. If yes, what are those factors?

**S2: Vignettes**

Introduction

A woman after KT of fertile age visits your outpatient clinic. She has a wish to conceive and therefore she visits you, for counseling and pregnancy advice. She has never been pregnant before. She was in need for a KT because she suffered from chronic glomerulonephritis. She received a KT from a living donor two years ago. Besides her KT and hypertension, she has no other comorbidities. Her BMI is 24. She is currently using tacrolimus 3 mg, twice a day and azathioprine 75 mg, once a day. Her hypertension is treated with amlodipine, once a day 10 mg.

**Vignette 1:** Her last measured blood pressure is 120/80 mmHg. eGFR is stable around 65 ml/min. In the urine samples there is a protein level of 0.2 gram/24 hrs. In the past two years there were no signs of rejection of the kidney transplant.

**Vignette 2:** Her last measured blood pressure is 120/80 mmHg. eGFR is stable around 65 ml/min. In the urine samples there is a protein level of 1 gram/24 hrs. In the past two years there were no signs of rejection of the kidney transplant.

**Vignette 3:** Her last measured blood pressure is 145/95 mmHg, her blood pressure is treated with hydrochloorthiazide and nifedipine instead of amlodipine. eGFR is stable around 65 ml/min. In the urine samples there is a protein level of 0.2 gram/24 hrs. In the past two years there were no signs of rejection of the kidney transplant.

**Vignette 4:** Her last measured blood pressure is 120/80 mmHg. eGFR is stable around 40 ml/min. In the urine samples there is a protein level of 0.6 gram/24 hrs. In the past two years there were no signs of rejection of the kidney transplant.

**Vignette 5:** Her last measured blood pressure is 140/95 mmHg. eGFR is about 25 ml/min. In the urine samples there is a protein level of 2 gram/24 hrs. In the past year, she has had an acute cellular rejection which was treated with methylprednisolone.

Questionnaire at each vignette

1. Do have a predominantly positive or negative stance towards a pregnancy for this patient?
2. Which of the factors below, and in what rating of importance, did you take into account in your positive of negative point stance towards a pregnancy for this patient? Please rate each factor from 1 (not important) to 5 (very important).

*a. Blood pressure*

*b. Proteïnuria*

*c. Preconceptional eGFR*

*d. Rejection*

1. If the woman in this vignette would become pregnant, what outcome would you expect regarding gestational age?

*a. <34 weeks*

*b. 34-37 weeks*

*c. >37 weeks*

1. If the woman in this vignette would become pregnant, what outcome would you expect regarding birthweight?

*a. 500-1000 gram*

*b. 1500-2000 gram*

*c. 2000-3000 gram*

*d. 3000-4000 gram*

1. If the woman in this vignette would become pregnant, what outcome would you expect regarding preeclampsia?

*a. 0-10%*

*b. 10-30%*

*c. 30-50%*

1. If the woman in this vignette would become pregnant, what outcome would you expect regarding graft loss within two years after pregnancy?

*a. 0-25%*

*b. 25-50%*

*c. 50-75%*

*d. 75-100%*

**S3: Checklist for Reporting Results of Internet E-Surveys (CHERRIES)**

| ***Checklist Item*** | ***Explanation*** | ***Page Number*** |
| --- | --- | --- |
| Describe survey design | Describe target population, sample frame. Is the sample a convenience sample? (In “open” surveys this is most likely.)  **Nephrologists and gynaecologists practicing in public hospitals were invited to participate** | 5 |
| IRB approval | Mention whether the study has been approved by an IRB.  **The study was approved by the Ethical Committee of the Erasmus Medical Centre: MEC-2020-0194.** | 7 |
| Informed consent | Describe the informed consent process. Where were the participants told the length of time of the survey, which data were stored and where and for how long, who the investigator was, and the purpose of the study?  **The length of the study, the investigator and the purpose of the study was described.** |  |
| Data protection | If any personal information was collected or stored, describe what mechanisms were used to protect unauthorized access.  **Personal information was collected and stored in a protected database, and were protected for unauthorized access by multiple passwords** | 7 |
| Development and testing | State how the survey was developed, including whether the usability and technical functionality of the electronic questionnaire had been tested before fielding the questionnaire.  **The case-vignette tool was constructed according to several steps. First, vignettes were designed based on previous literature and clinical expertise. Then, vignettes were evaluated by two experienced specialists in counselling for pregnancy after KT: one obstetrician and one transplant nephrologist. The vignettes were then reviewed by a health psychologist involved in survey research, to check for clear wording and corresponding questions. Finally, a study pilot was conducted by sending the survey to three transplant professionals to test replicability. According to these responses, the vignettes and questions were revised.** | 6 |
| Open survey versus closed survey | An “open survey” is a survey open for each visitor of a site, while a closed survey is only open to a sample which the investigator knows (password-protected survey).  **It is a closed survey password-protected** |  |
| Contact mode | Indicate whether or not the initial contact with the potential participants was made on the Internet. (Investigators may also send out questionnaires by mail and allow for Web-based data entry.)  **The initial contact was made by email. It was only possible to fill in the questionnaire if you have received a personal invitation by email.** |  |
| Advertising the survey | How/where was the survey announced or advertised? Some examples are offline media (newspapers), or online (mailing lists – If yes, which ones?) or banner ads (Where were these banner ads posted and what did they look like?). It is important to know the wording of the announcement as it will heavily influence who chooses to participate. Ideally the survey announcement should be published as an appendix.  **The survey was only send out by email, and announcements were made by scientific presentations** | 5 |
| Web/E-mail | State the type of e-survey (eg, one posted on a Web site, or one sent out through e-mail). If it is an e-mail survey, were the responses entered manually into a database, or was there an automatic method for capturing responses?  **The email was sent out through email. The responses were automatic captured in the protected Limesurvey database** | 5 |
| Context | Describe the Web site (for mailing list/newsgroup) in which the survey was posted. What is the Web site about, who is visiting it, what are visitors normally looking for? Discuss to what degree the content of the Web site could pre-select the sample or influence the results. For example, a survey about vaccination on a anti-immunization Web site will have different results from a Web survey conducted on a government Web site  **The survey was not linked to a website**. |  |
| Mandatory/voluntary | Was it a mandatory survey to be filled in by every visitor who wanted to enter the Web site, or was it a voluntary survey?  **The survey was not mandatory** |  |
| Incentives | Were any incentives offered (eg, monetary, prizes, or non-monetary incentives such as an offer to provide the survey results)?  **There were no incentives offered.** |  |
| Time/Date | In what timeframe were the data collected?  **A cross sectional survey vignette study was conducted between March 2020 - March 2021** |  |
| Randomization of items or questionnaires | To prevent biases items can be randomized or alternated.  **The questionnaire was not randomized** |  |
| Adaptive questioning | Use adaptive questioning (certain items, or only conditionally displayed based on responses to other items) to reduce number and complexity of the questions.  **We used adaptive questioning, certain items were displayed on responses to other items.** |  |
| Number of Items | What was the number of questionnaire items per page? The number of items is an important factor for the completion rate.  **Page 1: demographic questions, 17 questionnaire items (demographic questions)**  **Page 2-6: vignettes, 7 questions per vignette**  **Page 7: general questions on pregnancy after kidney transplantation, 5 questions** |  |
| Number of screens (pages) | Over how many pages was the questionnaire distributed? The number of items is an important factor for the completion rate.  **7 pages** |  |
| Completeness check | It is technically possible to do consistency or completeness checks before the questionnaire is submitted. Was this done, and if “yes”, how (usually JAVAScript)? An alternative is to check for completeness after the questionnaire has been submitted (and highlight mandatory items). If this has been done, it should be reported. All items should provide a non-response option such as “not applicable” or “rather not say”, and selection of one response option should be enforced.  **We used a completeness check in this questionnaire, people could only get forward if the mandatory questions were answered.** |  |
| Review step | State whether respondents were able to review and change their answers (eg, through a Back button or a Review step which displays a summary of the responses and asks the respondents if they are correct).  **Respondents were able to review and change their answers using a back button or a review step.** |  |
| Unique site visitor | If you provide view rates or participation rates, you need to define how you determined a unique visitor. There are different techniques available, based on IP addresses or cookies or both.  **Because the questionnaire was only available on personal invitation, we know the exact participation rates** |  |
| View rate (Ratio of unique survey visitors/unique site visitors) | Requires counting unique visitors to the first page of the survey, divided by the number of unique site visitors (not page views!). It is not unusual to have view rates of less than 0.1 % if the survey is voluntary.  **93 visitors visited the first page of the survey, because it was a closed survey this was only possible by clicking on the link.** |  |
| Participation rate (Ratio of unique visitors who agreed to participate/unique first survey page visitors) | Count the unique number of people who filled in the first survey page (or agreed to participate, for example by checking a checkbox), divided by visitors who visit the first page of the survey (or the informed consents page, if present). This can also be called “recruitment” rate.  **93 unique visitors visited the first page of the survey and 77 unique visitors filled in the first survey page**  **93/77 = 1.21** |  |
| Completion rate (Ratio of users who finished the survey/users who agreed to participate) | The number of people submitting the last questionnaire page, divided by the number of people who agreed to participate (or submitted the first survey page). This is only relevant if there is a separate “informed consent” page or if the survey goes over several pages. This is a measure for attrition. Note that “completion” can involve leaving questionnaire items blank. This is not a measure for how completely questionnaires were filled in. (If you need a measure for this, use the word “completeness rate”.)  **77 unique visitors fill in the first survey page and 62 completed all the questions.**  **77/62 = 1.24** |  |
| Cookies used | Indicate whether cookies were used to assign a unique user identifier to each client computer. If so, mention the page on which the cookie was set and read, and how long the cookie was valid. Were duplicate entries avoided by preventing users access to the survey twice; or were duplicate database entries having the same user ID eliminated before analysis? In the latter case, which entries were kept for analysis (eg, the first entry or the most recent)?  **No cookies were used for this survey.** |  |
| IP check | Indicate whether the IP address of the client computer was used to identify potential duplicate entries from the same user. If so, mention the period of time for which no two entries from the same IP address were allowed (eg, 24 hours). Were duplicate entries avoided by preventing users with the same IP address access to the survey twice; or were duplicate database entries having the same IP address within a given period of time eliminated before analysis? If the latter, which entries were kept for analysis (eg, the first entry or the most recent)?  **Because the invitations were personal , so duplicate entries were not possible** |  |
| Log file analysis | Indicate whether other techniques to analyze the log file for identification of multiple entries were used. If so, please describe.  **This was not used** |  |
| Registration | In “closed” (non-open) surveys, users need to login first and it is easier to prevent duplicate entries from the same user. Describe how this was done. For example, was the survey never displayed a second time once the user had filled it in, or was the username stored together with the survey results and later eliminated? If the latter, which entries were kept for analysis (eg, the first entry or the most recent)?  **All entries were kept for analysis. The most complete entries were kept for analysis** |  |
| Handling of incomplete questionnaires | Were only completed questionnaires analyzed? Were questionnaires which terminated early (where, for example, users did not go through all questionnaire pages) also analyzed?  **All questionnaires were analyzed. Also the ones which were terminated earlier. See also the flow chart of the study.** |  |
| Questionnaires submitted with an atypical timestamp | Some investigators may measure the time people needed to fill in a questionnaire and exclude questionnaires that were submitted too soon. Specify the timeframe that was used as a cut-off point, and describe how this point was determined.  **Because the nature of the questionnaire it was not possible to submit the questionnaire too soon.** |  |
| Statistical correction | Indicate whether any methods such as weighting of items or propensity scores have been used to adjust for the non-representative sample; if so, please describe the methods.  **We did not use propensity scores. We did adjust for multiple testing by using the Bonferroni method** |  |

This checklist has been modified from Eysenbach G. Improving the quality of Web surveys: the Checklist for Reporting Results of Internet E-Surveys (CHERRIES). J Med Internet Res. 2004 Sep 29;6(3):e34 [erratum in J Med Internet Res. 2012; 14(1): e8.]. Article available at [https://www.jmir.org/2004/3/e34](https://www.jmir.org/2004/3/e34/)/; erratum available <https://www.jmir.org/2012/1/e8/>. Copyright ©Gunther Eysenbach. Originally published in the [Journal of Medical Internet](http://www.jmir.org) Research, 29.9.2004 and 04.01.2012.

This is an open-access article distributed under the terms of the Creative Commons Attribution License (<https://creativecommons.org/licenses/by/2.0/>), which permits unrestricted use, distribution, and reproduction in any medium, provided the original work, first published in the Journal of Medical Internet Research, is properly cited.
